# High-Resolution Melting Analysis in Comparison with Microscopic Method: An Experimental Study to Diagnosis of *Plasmodium* Species Infections in Human

**DOI:** 10.18502/ijpa.v15i3.4205

**Published:** 2020

**Authors:** Mahya ALLAHMORADI, Afsaneh MOTEVALLI HAGHI, Mehdi NATEGHPOUR, Mehdi MOHEBALI, Ahmad RAEISI, Ahmad HOSSEINI SAFA, Sina MOHTASEBI, Mohammad Javad ABBASZADEH AFSHAR

**Affiliations:** 1. Department of Medical Parasitology and Mycology, School of Public Health, Tehran University of Medical Sciences, Tehran, Iran; 2. Center for Research of Endemic Parasites of Iran (CREPI), Tehran University of Medical Sciences, Tehran, Iran

**Keywords:** Malaria, Diagnosis, Microscopy, *Plasmodium*

## Abstract

**Background:**

Among the human parasitic diseases, malaria is the main cause of morbidity and mortality. To prevent the high mortality and tracking malaria elimination efforts, a prompt and sensitive diagnosis is essential. This study aimed to compare High-Resolution Melting (HRM) and microscopic methods to diagnose *Plasmodium falciparum* and *P. vivax*.

**Methods:**

Eighty-one blood samples were collected from patients with clinical symptoms who were suspect to malaria in Chabahar district, southeastern Iran and also, from those who were referred to Malaria National Laboratory in the Tehran University of Medical Sciences, Tehran, Iran. Microscopic examination and HRM method were used to the diagnosis of *Plasmodium* parasites simultaneously.

**Results:**

Microscopic results revealed 45 positive cases (12 *P. falciparum* and 33 *P. vivax*) out of 81 collected samples while according to HRM analysis results 11 and 33 samples were identified as *P. falciparum* and *P. vivax*, respectively. HRM analysis also revealed 1 mixed infection of *P. falciparum* and *P. malariae.*

**Conclusion:**

HRM analysis provides a promising mean for simultaneous detection and discrimination of the *Plasmodium* spp. especially in mixed infection cases.

## Introduction

Among the human parasitic diseases, malaria is the main cause of morbidity and mortality, with an estimated 219 million occurred cases and 435,000 mortalities around the world, in 2017 (1). Malaria infection, particularly in malignant forms, is one of the life-threatening diseases among the parasitic infections which can be transmitted mainly by mosquitoes belonging to the genus *Anopheles* in the most tropical and subtropical areas around the world (2, 3). *Plasmodium falciparum, P. vivax*, *P. malariae*, *P. ovale* and *P. knowlesi* are the main malaria species that infect humans of which, the first two, are endemic in southeastern Iran (4).

There are nonspecific symptoms in malaria patients such as chill, fever, sweating, anemia, splenomegaly, and some others that are similar to many infectious diseases. In order to prevent the mortality in children, especially those infected with *P. falciparum*, as well as for tracking malaria elimination efforts and investments, a prompt and sensitive diagnosis is essential (1, 2). The gold standard diagnostic method of malaria in humans is traditionally based on the microscopic detection of *Plasmodium* parasites in blood smears. This method is the most commonly used due to its low cost and simplicity. Besides the useful advantages for malaria microscopy there are some disadvantages such as requirement of skilled personnel, long processing times and low sensitivity in terms of low parasitemia infections (between 10–30 parasites/μL) and this is while molecular techniques offer detection of parasites at lower concentration of 5 parasites/μL and mixed malaria infections, as well as identification of the *Plasmodium* spp. (3). However, simultaneous application of both microscopic and molecular methods are recommended for the more accurate diagnosing process (1, 5).

Among the molecular techniques, High-Resolution Melting (HRM) analysis is an automated and analytical method which monitors the fluorescence emitted during the reaction as an indicator of amplicon production at each PCR cycle (in real time), as opposed to the endpoint detection. Also, due to being a closed tube method, it does not bear any contamination (6, 7). The HRM analysis procedure involves amplification of the region of interest in the presence of a specialized dsDNA binding dye and gradually denaturation of amplicons by increasing the temperature in small increments in order to produce a characteristic melting profile that is called melting analysis (2).

In malaria malignant forms, misdiagnosis may lead to death in the patients (1). Therefore, new and more accurate diagnosis methods would continuously be developed. The present study was conducted to compare HRM analysis with the microscopic method in detection and identification of *P. falciparum* and *P. vivax* that are predominant in malaria endemic areas of Iran.

## Materials and Methods

### Blood sample collection

**Fig. 1:**
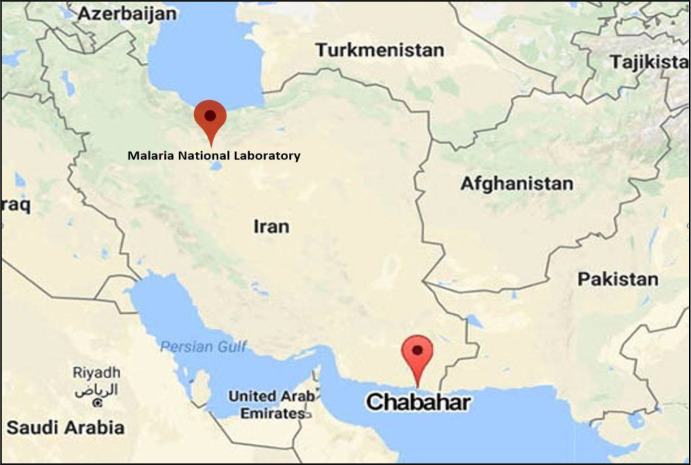
Chabahar district and Malaria National Laboratory in Tehran, Iran

The blood samples were collected from 81 patients with clinical symptoms who were suspected to malaria in Chabahar district, southeastern Iran neighboring Pakistan and also from those who were referred to Malaria National Laboratory in the School of Public Health, Tehran University of Medical Sciences, Tehran, Iran from 2014 to 2016 (Fig. 1).

Previous to sampling a written consent was obtained from each of the participants according to the Research Ethical Protocol of Tehran University of Medical Sciences.

A pre-designed checklist including demographic factors also was filled out from all of the enrolled patients. An amount of 2 mL blood was obtained via venipuncture into EDTA treated tube from each of the enrolled patients. Prior to any more process, two Giemsa stained thick and thin smears were prepared for microscopic examination and the rest of the blood samples were frozen at −20 °C until relevant test processes.

### Microscopic examination

Microscopic examination was performed based on WHO guideline (8). Briefly, thin and thick blood smears were stained with 5% (vol/vol) Giemsa stain in distilled water (pH~7.2) and examined under the light microscope for the presence of malaria parasites using 1000× magnification with immersion oil. Detecting either *P. vivax* or *P. falciparum* in each blood film was called as positive and those were defined as negative if no parasite was observed in 300 microscopic fields. Also, detecting two species of the parasite in one blood film was considered as a mixed infection.

### DNA Extraction

Total DNA was extracted from 200 μL of the blood sample using MACHEREYNAGEL (MN) DNA isolation kit (Germany), following the manufacturer’s instructions.

### DNA amplification and HRM analysis

The 18S SSU rRNA gene was amplified using specific primers (forward primer-5′ GRAACTSSSAACGGCTCATT-3′ and reverse primer-5′-ACTCGATTGATACACACTA-3′) (2). The conventional PCR was carried out according to Chua et al. protocol (2). Briefly; 25 μL final reaction volume was prepared to contain 10 μL Master Mix 2× (Ampliqon, Denmark), 9 μL distilled water, 1 μL of each primer, and 4 μL of template DNA. PCR was performed using the following cycling protocol: an initial denaturation step at 95 °C for 10 min, followed by 35 cycles at 95 °C for 30 s, 59 °C for 30 s, 72 °C for 30 s, and a final extension step at 72 °C for 5 min. Also, a negative control was applied (distilled water) in each run.

For HRM analysis the kit contained the novel double-stranded DNA-binding fluorescent dye, EvaGreen, and an optimized HRM PCR master mix buffer, consisting of HOT FIREPol DNA Polymerase, ultrapure dNTPs, MgCl_2_ and EvaGreen dye (Solis Biodyne, Estonia) was used. HRM temperature was raised from 65 °C to 85 °C. During this process, the amplicons obtained from the previous PCR were denatured prior to the development of melting curves in the inflexion point where changes in fluorescence with respect to changes in temperature (Df/Dt) were recorded with a ramp of 0.3 °C/sec (9) and Fluorescence dye signaling was measured after each cycle. Standard positive controls were used for *P. falciparum* and *P. vivax* available in the Department of Medical Parasitology and Mycology, School of Public Health, Tehran University of Medical Sciences.

Real-time PCR was carried out in a Mini Opticon real-time PCR detection system (Applied Biosystems Step One Plus Inc., CA, USA). The real-time amplification result and T_m_ analysis were obtained using the Step One Plus^TM^ software version 2.3 (Life technologies).

T_m_ analysis was repeated three times in each run to confirm the repeatability of the T_m_ assay by estimating the T_m_ variation within a PCR amplification (intra-assay), and between PCR amplifications (inter-assay). The coefficient of variation (CV) was calculated by dividing the standard deviation (SD) by the arithmetic mean of the measured values of T_m_ (CV = SD [Le, 2012 #11]/mean value).

Also, to check the uniformity of temperature in the cycler block, a number of samples were re-amplified at different positions of the cycler block during the same amplification cycle. The intra-assay CVs represent the mean CVs of the results obtained from the replications of *Plasmodium* spp.

## Results

### Microscopic and HRM examinations

**Fig. 2:**
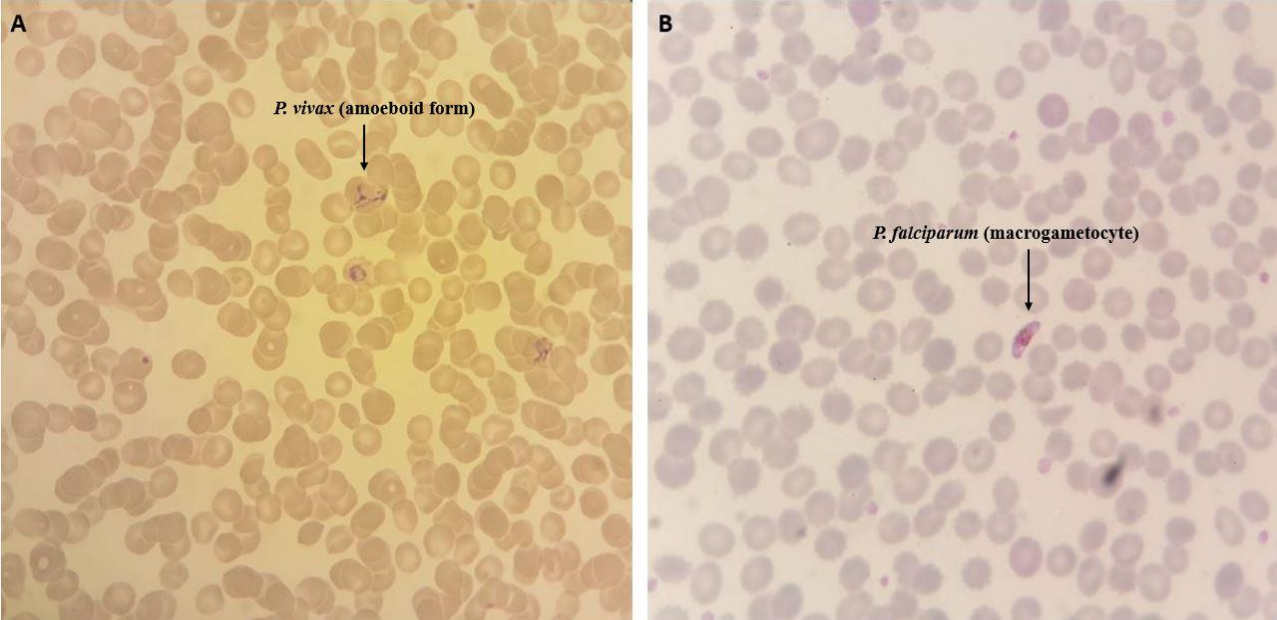
*P. vivax* (A) and *P. falciparum* (B) species identified in microscopic examination

**Fig. 3:**
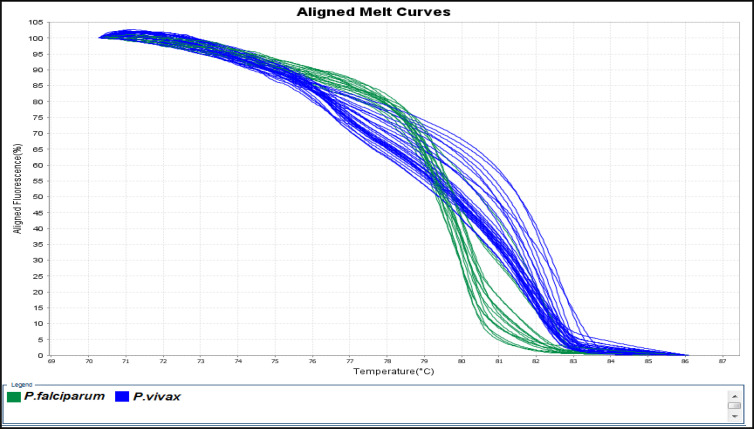
HRM based on (EvaGreen) Aligned Melt curves analyses and identified *Plasmodium* spp. using 18S SSU rRNA gene

**Fig. 4:**
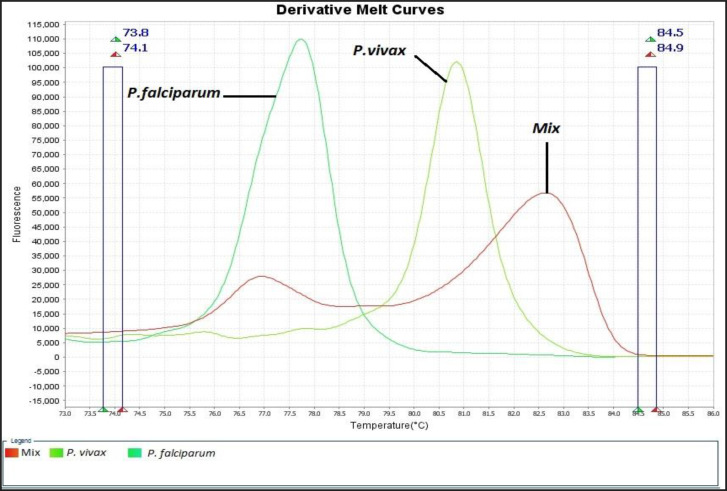
The melting curve of the sample with the mixed infection which shows two significant peaks related to *P. falciparum* and *P. malariae* species

**Fig. 5:**
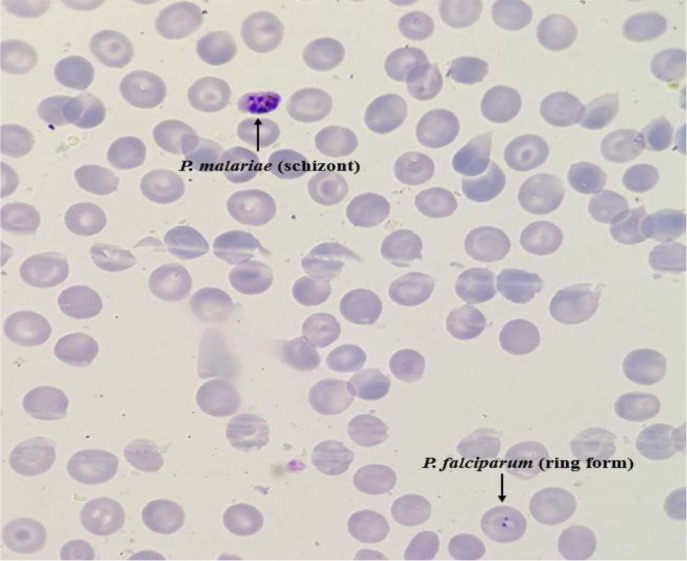
The mixed infection with *P. falciparum* and *P. malariae*

Among 81 samples which were examined for diagnosing malaria parasites with the standard microscopic method 45 cases including 12 *P. falciparum* and 33 *P. vivax* were diagnosed as positive cases and the rest remained as negative samples (Fig. 2). According to HRM analysis results, 11 and 33 samples were identified as *P. falciparum* and *P. vivax*, respectively. The melting curves of *P. falciparum* and *P. vivax* that were identified by sequencing process are shown in Fig. 3. Also, HRM analysis results revealed 1 mixed infection of *P. falciparum* and *P. malariae* (a patient with a history of traveling to Tanzania) which was proved in the detailed re-examination of the sample microscopically (Fig. 4 and 5). Assessment of intra- and inter-assay variability showed low and acceptable CVs. (Table 1).

**Table 1:** Mean T_m_, SD, and CV calculated based on intra- and inter-assay of 18S SSU rRNA gene sequence of *Plasmodium* spp.

***Gene*** ***18S SSU rRNA***	***Mean T_m_ (°C)***	***SD***	***Intra-assay CV (%)***	***Inter-assay CV (%)***
*P. falciparum*	77.70±0.2	0.12	0.10	0.12
*P. vivax*	79.77±0.2	0.14	0.13	0.14

T_m_: Melting temperature

## Discussion

In the present study, we have successfully utilized a real-time PCR assay coupled with HRM analysis on the 18S SSU rRNA gene for rapid, sensitive and reliable detection of *Plasmodium* spp*.* in comparison with the microscopic method as the gold standard. The average of T_m_ variation obtained by melting curve analysis was about 2°C, which reflect the fact that HRM has sufficient ability and reliability to discriminating *P. falciparum* and *P. vivax*.

Since the importance of malaria elimination in Iran (10), more comprehensive studies on the diagnosis of this disease and the identification of *Plasmodium* species are necessary to achieve this goal. It is known that the majority of epidemiological studies of *Plasmodium* infections are based on conventional microscopy as the diagnostic gold standard method. Although this method is technically simple and not costly, it is hampered by the fact that the technique is time-consuming and requires an experienced technician and also, low sensitivity in low parasitemia situations (11).

Molecular methods as sensitive tools are useful to identify malaria infections, particularly in low parasitemia and mixed-infection cases (12). HRM analysis is a completely closed tube assay that does not employ additional post-PCR steps; thus, the risk of contamination is low. In recent years, several molecular techniques, based on PCR have been developed for the specific identification and characterization of *Plasmodium* infection (13–16). Although these techniques are sensitive and specific for the identification of *Plasmodium* species, they are laborious and time-consuming, especially the post-PCR processing steps. In addition, there is also a higher risk of contamination, they are more expensive (e.g., DNA sequencing) and the techniques only provide qualitative information.

There are some studies used HRM for various purposes. It has been applied in the study of parasitic protozoa such as rapid detection of SNPs associated with antimalarial drug resistance in *P. falciparum* genes (17). In a study (18), this technique was applied to differentiate among the *Naegleria* species. Also, HRM has been applied for the differentiating *Leishmania* spp. in both human and animal samples (19). In addition, HRM has been used in parasitic helminth studies such as rapid differentiation between *Fasciola hepatica* and *F. gigantica* and also, *Echinococcus granulosus* and *E. canadensis* (20, 21).

The results of this study revealed both HRM analysis on the 18S SSU rRNA gene and microscopic method based on thick and thin blood smears are consistent in terms of *Plasmodium* spp. detection, however malaria patients may harbor multiple species of *Plasmodium* which in such situations, molecular methods, due to their greater sensitivity are far better than the microscopic method. In this study, an HRM curve with two peaks which represents a mixed infection was obtained in relation to one of the samples. One of the peaks complies with *P. falciparum* positive control and the other one was not matched with the positive controls of *P. falciparum* and *P. vivax* available in our laboratory. Hence, a detailed microscopic re-examination beyond the WHO standards was done which only two schizonts of *P. malariae* were seen in addition to *P. falciparum* forms in the blood smear.

Based on our results and also, Chua et al. study (2), HRM assay is an appropriate tool for the detection of mixed malaria infections. Nevertheless, some studies have reported a number of mixed malaria infections in humans via the utilization of conventional methods (14, 15, 22). For instance, 28.4% of the participant were harboring a mixed infection of *P. vivax* and *P. falciparum* by nested-PCR (23). It seems that HRM analysis, given its mentioned advantages over other methods, is a powerful alternative to these conventional molecular methods.

The current investigation met some limitations, including small sample size and lack of positive control for *P. malariae* in our laboratory.

## Conclusion

HRM analysis provides a promising molecular tool for simultaneous detection and discrimination of the *Plasmodium* spp. especially in mixed infection cases. It seems that, regarding the advantages over other methods, HRM can apply as a molecular epidemiology tool for rapid screening of large numbers of samples.
